# Logical operations with single x-ray photons via dynamically-controlled nuclear resonances

**DOI:** 10.1038/srep25136

**Published:** 2016-04-27

**Authors:** Jonas Gunst, Christoph H. Keitel, Adriana Pálffy

**Affiliations:** 1 Max-Planck-Institut für Kernphysik, Saupfercheckweg 1, D-69117 Heidelberg, Germany

## Abstract

Photonic qubits lie at the heart of quantum information technology, often encoding information in their polarization state. So far, only low-frequency optical and infrared photons have been employed as flying qubits, as the resources that are at present easiest to control. With their essentially different way of interacting with matter, x-ray qubits would bear however relevant advantages: they are extremely robust, penetrate deep through materials, and can be focused down to few-nm waveguides, allowing unprecedented miniaturization. Also, x-rays are resonant to nuclear transitions, which are very well isolated from the environment and present long coherence times. Here, we show theoretically that x-ray polarization qubits can be dynamically controlled by nuclear Mössbauer resonances. The control knob is played by nuclear hyperfine magnetic fields, that allow via fast rotations precise processing of single x-ray quanta polarization. With such rotations, single-qubit and binary logical operations such as a destructive C-NOT gate can be implemented.

The qubit is the quantum analogue of the classical bit, the unit of information^1^. It is typically embodied by a two-state quantum-mechanical system, which can, unlike its classical counterpart, exist in a superposition of both states at the same time, a property fundamental for quantum information technology. A typical example is the polarization of a single photon. Although polarization can be used to encode quantum information regardless of the photon frequency, so far in practice photonic qubits have been restricted to the optical regime^2^,^3^,^4^,^5^,^6^. Responsible for this is the exceptional level of control achieved in this frequency region, better than for x-rays and *γ*-rays. However, x-rays would be very attractive for quantum technology applications with their good detection efficiency, penetration power, and remarkable focus, reaching down to few nanometers at present^7^,^8^ and being in practice far from any diffraction-limit constraint. Admittedly, experimental challenges at the large coherent x-ray facilities today will require a different paradigm compared to optical laser experiments. Progress on the field of table-top x-ray sources based on laser acceleration^9^,^10^,^11^,^12^ opens however new possibilities for x-ray generation and manipulation in normal-size laboratories worldwide.

While atomic transitions are naturally used to resonantly manipulate optical photons, nuclear transitions may be the elementary counterparts for x-rays. Nuclear transitions present a clean, well isolated system robust against disturbances in their electronic environment with very long coherence times, lasting up to hundreds of nanoseconds or longer. This makes them ideal for x-ray quantum optics applications^13^,^14^, but also for quantum information processing, provided that efficient control mechanisms can be developed. Control at the single-photon level has been recently demonstrated in a laboratory-scale Mössbauer setup, where the coherent manipulation of waveforms of individual x-ray photons has been achieved^15^. Such control procedures operated at single-photon nuclear interfaces and progress in x-ray optics open the perspective to extend fields like quantum information and quantum communication to photon energies in the keV-range^16^.

Can polarization-encoded single x-ray photons be coherently processed by means of resonant nuclear interactions? We show in the following from the theory side that precise control and processing schemes for the polarization of individual x-rays can be achieved by fast rotations of the nuclear hyperfine magnetic field. Such rotations can dynamically manipulate the polarization response of the nuclei and allow the implementation of logical operations, the fundamental ingredients of quantum information. The system under investigation involves a nuclear solid-state sample with Mössbauer ^57^Fe nuclei in a nuclear forward scattering setup (Fig. 1). The ^57^Fe nucleus has a stable ground state and a first excited state at 14.4 keV (wavelength 0.86 Å), well in the x-ray regime. The sample is subject to a hyperfine magnetic field that splits the ground and first excited states according to their nuclear spin (inset in Fig. 1). Each of the six resulting transitions are very narrow, with natural widths of only few neV.

A resonant broadband x-ray pulse from a synchrotron radiation source propagates along the *y*-direction and impinges perpendicularly on the nuclear sample. The radiation is linearly polarized with *x*-(*z*-)polarized light denoted as *σ*-(*π*-)polarization by convention^17^. Due to the narrow nuclear resonances and the low brilliance of x-ray sources, at most one x-ray photon is resonant and only one nucleus can be excited in the sample. The recoilless nature of this transition in solid-state nuclear targets leads to the formation of a delocalized, collective excitation, in literature referred to as nuclear exciton^18^ or “timed” Dicke state^19^ which decays coherently into the forward direction leading to a relative speed-up and enhancement of the yield at the forward-placed detector. The excitation of the Dicke state strongly depends on the number of contributing nuclear scatterers and on the spectral photon flux over the resonant frequency window. With the broadband synchrotron pulse covering all six hyperfine transitions, the single resonant photon can drive simultaneously several polarization-selected hyperfine transitions, leading to quantum beats in the measured spectra. For instance, initially *σ*-(*π*-)polarized x-rays couple to all Δ*m* = 0 (Δ*m* = ±1) transitions provided the magnetic field ***B*** points along the *z*-direction. Since only those photons are coherently scattered into the forward direction for which the nucleus returns to its original ground state Zeeman level, the *σ*-(*π*-)polarization is conserved in the course of the scattering as long as the hyperfine magnetic field is held constant.

The situation changes if during the scattering process, the nuclear hyperfine magnetic field is rotated. Let us assume for instance that the magnetic field at the nuclear target ***B***_I_ is initially constant and points along the *z*-axis. A fast rotation of the magnetic field after the nuclear excitation has occurred leads to an almost instantaneous change of the quantization axis and a redistribution of the collective excitation among the Zeeman levels. In the subsequent decay process, interference effects may occur which can suppress the decay via certain polarization states. The key parameters here which directly determine the scattered x-ray photon polarization are the rotation geometry and the rotation instant *t*_0_ (see Methods). For an efficient translation of *σ* into *π* polarization and vice-versa, a 90° counterclockwise rotation around the *y* direction to ***B***_II_ parallel to the *x*-axis (Fig. 1) is most convenient.

For the implementation of logical operations with x-rays, switching instances *t*_0_ where *σ* and *π* polarizations are simultaneously converted into (pure) opposite polarization states need to be found. A first example are the four one-qubit logic gates Identity, True, False and Negation (Fig. 2b). The single-photon qubits can be encoded as x-ray orthogonal polarization states, for instance “0” as *π*- and “1” as *σ*-polarization. By means of a semi-classical wave equation for the x-ray field (see Methods) we can show that almost identical switching times exist converting the polarization states according to these truth tables (Fig. 2). For instance, a magnetic field rotation of 90° at *t*_0_ ≈ 22.3 ns corresponding to a local minimum of each individual quantum beat simultaneously converts *σ* into *π* and vice versa, successfully implementing Negation for all times *t* > *t*_0_. We quantify the success rate of the implemented x-ray gates by introducing the parameters *ε*, which gives a measure of the intensity loss at times *t* < *t*_0_, and *η* that describes the probability of realization for the x-ray gates for times *t* > *t*_0_ (see Methods). The switching moments *t*_0_ as given in Fig. 2 only become uniquely defined by setting the additional constraint to minimize *ε*. Numerical values for *ε* and *η* are provided in Table 1.

Limiting factors for the probability of realization are (i) small mixing of the “wrong” polarization state due to multiple scattering events that are not accounted for in our choice of the switching time *t*_0_; (ii) small mismatches between the switching times for input *σ* and *π* (for instance, for Negation, 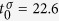 ns and 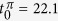 ns) and (iii) the accuracy of the experimental switching time. So far, most promising are ^57^Fe-enriched FeBO_3_ samples, for which magnetic field switching times of less than 4 ns were reported^20^,^21^. Choosing averaged switching times in between 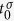 and 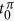, the calculated success rate *η* drops compared to the values presented in Table 1 but remains better than 95% for all four unary gates. Finally, the condition *t* > *t*_0_ strongly reduces the total probabilities of realization, since photons released before the time *t*_0_ defined by the vertical dashed line in Fig. 2 are lost. Two approaches depicted in Fig. 3 may circumvent the depicted limitations. First, one may introduce a polarization-sensitive time delay line^22^ such that the two switching times exactly match and losses are minimized. Alternatively, a polarizer can be used to spatially split the x-ray pulse in two parts that interact with two separated nuclear targets. The magnetic field rotations can be then chosen independently of each other and individually optimized leading to theoretical probabilities of realization *η* larger than 97%. We note that based on our results for pure polarization states, also superpositions thereof can be effectively processed in logical operations (see Supplementary Information).

With optimized one-qubit logical operations, we may now turn to the implementation of binary logical gates by means of x-ray photons. Since the x-ray-nuclear interface hosts a single photon only, a second, temporally synchronized photon is required in order to induce an effective nonlinearity as control. An artificial coupling can be introduced if the magnetic field rotation is triggered by detection of the second control photon (Fig. 1) which must not necessarily be on resonance to the nuclear transition, e.g. it could be in the optical range. The magnetic field rotation could be applied at a predetermined switching time *t*_0_ (counted from the incidence of the x-ray target pulse at *t* = 0), in case a control photon with the desired polarization is detected at the trigger. Alternatively, the detection event of the control photon may trigger a prompt rotation of the magnetic field.

Let us exemplify this on the example of the C-NOT gate, which flips the state of a target (T) qubit conditional on a control (C) qubit being in the logical state “1”^1^. The magnetic field rotation is applied with a predetermined switching time of 22.3 ns if a control photon with polarization *σ* (filtered by a polarizer) is detected at the trigger. Since the information associated with the control photon is destroyed during operation, and the polarization control relies on resonant scattering, this setup corresponds to a nondeterministic version of a destructive CNOT gate^23^,^24^, which cannot be used directly for reversible computing^25^. A non-destructive version would require harnessing quantum teleportation^26^ to transfer the polarization state of the detected control photon to another physical qubit.

A proof-of-principle experiment can be carried out already today at synchrotron radiation facilities which have access to the keV photon energy regime, high repetition rates, negligible sample damage and short pulses compared to the time-scale of the nuclear response (~ns). A fast triggering process is guaranteed by today’s photo-diodes which have response times shorter than 1 ns^27^. X-ray linear polarization can be measured with precision up to 0.3° using polarimeters based on the Compton effect^28^, and Bragg reflections on crystals can filter polarizations states as good as 10^−6^%^29^,^30^. Radioactive sources or x-ray parametric down conversion^31^ may provide alternative sources for single x-ray photons for quantum information processes. Experiments at novel x-ray free electron sources^32^,^33^,^34^ may facilitate with their high brightness and coherence in the future the implementation of binary non-destructive x-ray gates by allowing both target and control photons to be on resonance to the nuclear transition.

## Methods

Theoretically, we describe the coherent nuclear scattering process by a semi-classical wave equation^21^. The x-ray electric field in front and behind the target can be written as a time-modulated plane wave ***E***(*y*, *t*)e^*i*(*ky*−*ωt*)^. The calculation of the scattered field amplitude behind the target is carried out within the slowly-varying envelope approximation using perturbation theory and can be written as a summation over all multiple scattering orders *p* from 1 to ∞. The incident pulse *p* = 0 is not of interest here and is typically eliminated in experiments by means of time gating. In a first approximation, all multiple scattering events are assumed to occur only before the magnetic field switching, leading to the following expression for the electric field


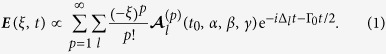


**Figure 2:**
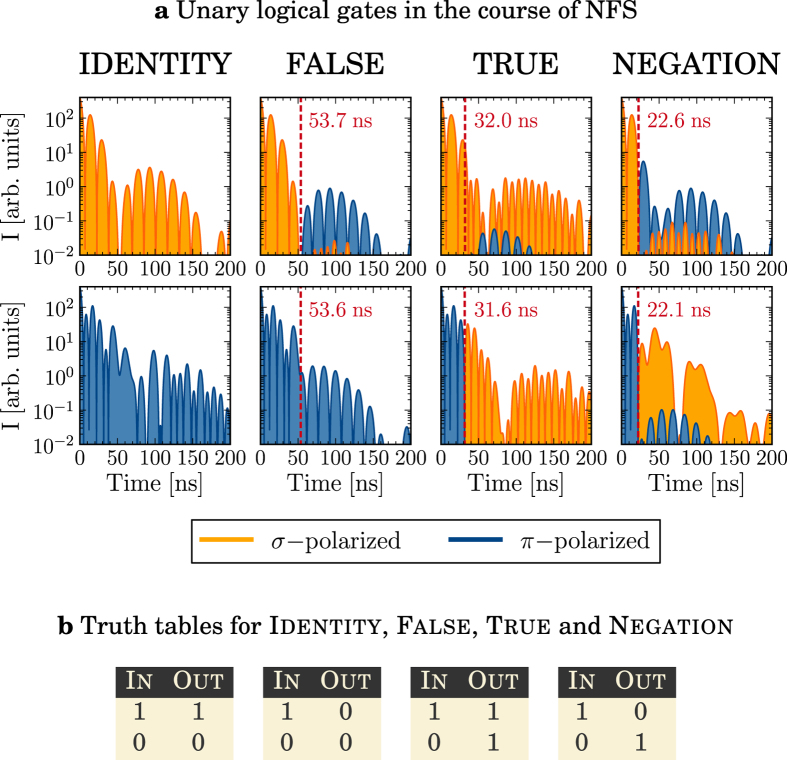
One-qubit logical operations with x-rays. (**a**) Nuclear scattering intensity spectra with an optical depth of *ξ* = 10 are shown for initially *σ*- (top row) and *π*–polarized (bottom row) x-rays. The switching times *t*_0_ (red dashed lines) determine the implemented logical operation. (**b**) Corresponding truth tables.

Here, *ξ* is the optical depth of the medium, Δ_*l*_ describes the detuning from the nuclear transition frequency *ω*_0_ due to magnetic hyperfine splitting and Γ_0_ represents the natural transition width. For each contributing nuclear transition *l* between the hyperfine-split levels and scattering order *p*, the time-independent amplitudes 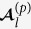 are completely determined by the magnetic field rotation geometry via the Euler angles *α*, *β* and *γ* and by the switching time *t*_0_. The expression (1) represents the dominating contribution to the scattered field and can be used to determine up to a good approximation the desired switching parameters. By changing the order of the summations, the scattered radiation via the nuclear transition *l* can be expressed as a product between a sum of time-independent amplitudes and a time-dependent phase factor, with specific parameter sets for which constructive or destructive interference between the summation terms with different *l* occur. A suitable choice of *t*_0_, *α*, *β* and *γ* can control the scattered photon polarization on single-photon nuclear interfaces, building the basis for the compilation of logical x-ray gates. The numerical results for the scattered field are obtained going beyond the approximation in Eq. (1) to include all multiple scattering events before and after *t*_0_. The sum over the scattering order *p* converges quickly such that including the first 14 scattering orders (*p*_max_ = 14) is already sufficient for *ξ* = 10. The field intensity (Fig. 2) is then proportional to |***E***(*ξ*, *t*)|^2^.

In order to quantify the success rate of the implemented x-ray gates, the measures *ε* and *η* are introduced via


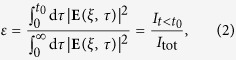


and


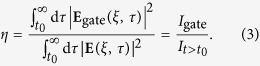


The quantity *ε* in Eq. (2) is defined as the integrated intensity for times smaller than the moment of switching 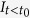 divided by the total integrated intensity *I*_tot_. Since the gate operation can be only realized for *t* > *t*_0_, *ε* gives a measure of the intensity loss at times *t* < *t*_0_. Due to multiple scattering events that cannot be accounted for in the choice of *t*_0_, the scattered radiation field after *t*_0_ also contains small distortions from the “wrong” polarization output. The quantity *η* describes the probability of realization for the x-ray gates for times *t* > *t*_0_. In Eq. (3), ***E***_gate_ corresponds to the polarization component of a successful gate operation, e.g., in the case of the TRUE: ***E***_gate_ = (***E***⋅***e***_*σ*_)***e***_*σ*_.

## Additional Information

**How to cite this article**: Gunst, J. *et al.* Logical operations with single x-ray photons via dynamically-controlled nuclear resonances. *Sci. Rep.* **6**, 25136; doi: 10.1038/srep25136 (2016).

## Supplementary Material

Supplementary Information

## Figures and Tables

**Figure 1:**
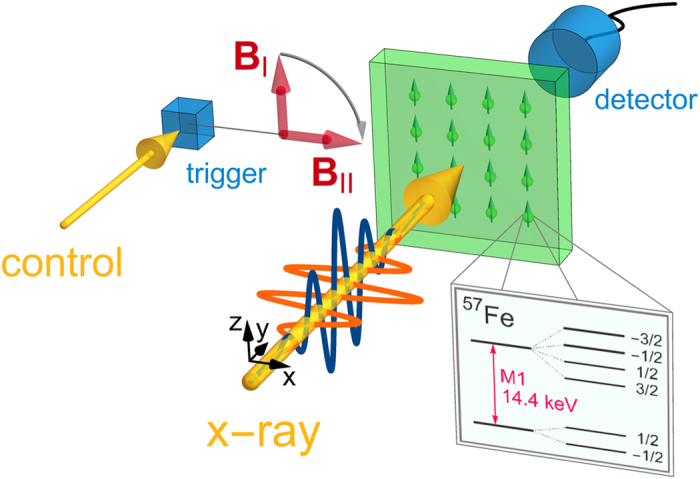
Nuclear forward scattering setup. *σ*- (orange, lighter hue) or *π*-polarized (blue, darker hue) x-rays scatter off a nuclear target in the forward direction. A spatially separated control photon triggers a magnetic field rotation from the *z*- to the *x*-axis. The hyperfine-split nuclear level scheme of ^57^Fe is illustrated in the inset.

**Figure 3:**
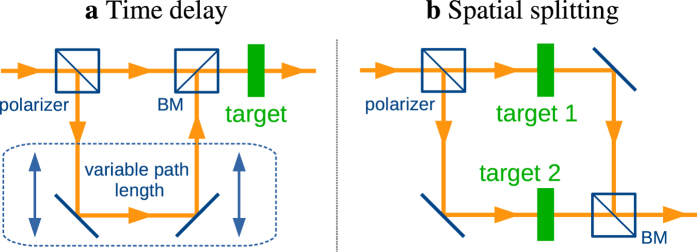
Alternative setups for efficient implementation of logical operations. (**a**) A time delay is introduced for one of the polarization components by means of polarizers^29^,^30^, beam mixers (BM)^22^ and x-ray mirrors^35^. (**b**) Two separated nuclear targets are used such that for each polarization component, the optimal switching *t*_0_ can be implemented independently.

**Table 1:** Loss rate *ε* and probability of realization *η* for the case of optimized switching moments as used in Fig. 2.

	Identity	False	True	Negation
*ε^σ^* (%)	0	95.2	93.5	87.4
*ε^π^* (%)	0	92.5	82.9	73.6
*η*^*σ*^ (%)	100	97.0	98.3	95.9
*η*^*π*^ (%)	100	99.4	99.9	99.3

Superscripts *σ* and *π* refer to initial polarization state of the incoming radiation.
