# Supplementary material for: Logical operations with single x-ray photons via dynamically-controlled nuclear resonances
Source: Sci Rep. 2016 Apr 27;6:25136. doi: 10.1038/srep25136 (PMC4846863; doi:10.1038/srep25136)
Supplement: Supplementary Information [file srep25136-s1.pdf]

Supplementary information to "Logical operations with single x-ray photons via dynamically-controlled nuclear resonances"

Jonas Gunst, Christoph H. Keitel and Adriana Pálffy
Max-Planck-Institut für Kernphysik, Saupfercheckweg 1, 69117 Heidelberg, Germany

COMMENT ON DIVINCENZO'S CRITERIA FOR QUANTUM COMPUTING [S1]

1. *A scalable physical system with well characterized qubits*

In our approach, the polarization of single photons is used in order to encode information. The following qubit notation is adopted: $|0\rangle = \pi$, $|1\rangle = \sigma$. The scalability of x-ray photonic qubits is similar to the one of optical qubits, with the difference that potentially, x-ray photons can be much tighter focused than their longer-wavelength counterparts. For instance, a 7 nm focus has been recently achieved [7]. In conjunction with 10-nm x-ray waveguides [8], focusing might bring a significant improvement in the spatial scalability of x-ray qubits.

2. *The ability to initialize the state of the qubits to a simple fiducial state, such as* $|000\ldots\rangle$

For photonic qubits the initialization goes back to a suitable single-photon source. In the keV energy regime, x-ray parametric down-conversion [31, S2–S4] is a way to construct a heralded single x-ray photon source. In this process a non-linear crystal (typically diamond) is used to split up an incoming pump x-ray into an idler and a signal photon. Detection of the idler photon heralds the emission of the signal single photon. As an alternative, in this work we envisage a proof-of-principle experiment that uses the low spectral density of synchrotron radiation (SR) pulses to construct a single-photon x-ray source. The SR pulse drives a narrow-width nuclear transition in ^{57}Fe. Typically, at most one single photon in the SR pulse is resonant to the nuclear transition. The resonant photon will be filtered out by the nuclear transition and re-emitted as a single quantum long after the rest of the SR pulse has left the setup.

3. *Long relevant decoherence times, much longer than the gate operation time*

In general, photonic qubits have very long coherence times. Moreover, the loss rates are low when moving in free space. The magnetic field rotations envisaged in this work take place on a nanosecond scale, while the coherence time is on the order of 100 ns.

4. *A universal set of quantum gates*

For a universal set of quantum gates, single- and two-qubit operations are required. Since photons do not interact with each other in free space, two-qubit operations are difficult to realize. Here, we have shown that logical operations on single x-ray photons can be simulated by using nuclear interfaces. Moreover, an effective photon-photon interaction has been artificially introduced by correlating the gate operation with the detection time of the control photon in order to realize a destructive version of the two-qubit CNOT gate. The basic requirement for the realization of the two-qubit CNOT gate is that the single-qubit gates can be operated at a single nuclear target solely by varying the magnetic field rotation instant.

5. *A qubit-specific measurement capability*

The polarization of x-rays can be measured by instruments based on the principle of Compton and Rayleigh polarimetry. For instance, Ge detectors have already been successfully applied in a photon energy range between 30 and 100 keV. A polarization resolution of $0.3°$ has been achieved [28]. In the case of smaller wavelengths it is favorable to use Si-PIN diodes instead of Ge detectors since less background radiation is collected. Concerning the 14.4 keV x-ray photons emitted from the nuclear decay of the first excited state in ^{57}Fe, it is additionally possible to use high-precision polarizers. These polarizers are based on successive reflections in so-called channel cut crystals which results in a polarization purity of more than 9 orders of magnitude [30]. The realization of such a high polarization purity paves the way for measuring tiny changes of the photon polarization state up to a few arcsec [30]. After separation by the polarizer, the single x-ray photons can be detected, time-resolved, with fast avalanche photo-diodes, which have approx. 1 ns time resolution [27].

APPLICATION TO SUPERPOSITION STATES

By applying timed rotations of an external magnetic field, single-photon nuclear interfaces can be used in order to simulate logical operations. Initially either π- or σ-polarized x-rays have been considered. According to the introduced qubit notation and by using Eq. (1), the

following gate transformations are realized:

$$\begin{aligned}
\text{identity:}\quad & |0\rangle \to \psi^0_{id}(t)\,|0\rangle\,, |1\rangle \to \psi^1_{id}(t)\,|1\rangle\,, \\
\text{false:}\quad & |0\rangle \to \psi^0_{false}(t)\,|0\rangle\,, |1\rangle \to \psi^1_{false}(t)\,|0\rangle\,, \\
\text{true:}\quad & |0\rangle \to \psi^0_{true}(t)\,|1\rangle\,, |1\rangle \to \psi^1_{true}(t)\,|1\rangle\,, \\
\text{negation:}\quad & |0\rangle \to \psi^0_{neg}(t)\,|1\rangle\,, |1\rangle \to \psi^1_{neg}(t)\,|0\rangle\,. \qquad \text{(S1)}
\end{aligned}$$

Here, the time-dependent amplitudes ψ describe the resonant scattering process after the magnetic field rotation ($t > t_0$). They are determined via Eq. (1). The small perturbations from the "wrong" polarization state (see Fig. 2) have been neglected in Eqs. (S1). For an initial single x-ray photon in a superposition state $|\Psi\rangle = \alpha\,|0\rangle + \beta\,|1\rangle$ with $\alpha, \beta \in \mathbb{C}$, the gate transformation properties can be straightforwardly generalized to

$$\begin{aligned}
\text{identity:}\quad & |\Psi\rangle \to \alpha\,\psi^0_{id}(t)\,|0\rangle + \beta\,\psi^1_{id}(t)\,|1\rangle\,, \\
\text{false:}\quad & |\Psi\rangle \to \alpha\,\psi^0_{false}(t)\,|0\rangle + \beta\,\psi^1_{false}(t)\,|0\rangle\,, \\
\text{true:}\quad & |\Psi\rangle \to \alpha\,\psi^0_{true}(t)\,|1\rangle + \beta\,\psi^1_{true}(t)\,|1\rangle\,, \\
\text{negation:}\quad & |\Psi\rangle \to \alpha\,\psi^0_{neg}(t)\,|1\rangle + \beta\,\psi^1_{neg}(t)\,|0\rangle\,. \qquad \text{(S2)}
\end{aligned}$$

Note that the scattering amplitudes ψ occurring in Eqs. (S2) may imprint an additional time-dependent phase between $|0\rangle$ and $|1\rangle$ because different hyperfine transitions are involved depending on the initial polarization state and/or on the nature of the induced gate transformation. The time dependence of this phase difference is well-known but may be difficult to control in the same operation.

After the gate transformations, high-precision polarizers can be used to filter out and detect the pure polarization states $|0\rangle$ and $|1\rangle$.

[S1] DiVincenzo, D. P. The physical implementation of quantum computation. *Fortschr. Phys.* **48,** 771–783 (2000).

[S2] Tamasaku, K., Sawada, K. Nishibori, E. & Ishikawa, T. Visualizing the local optical response to extreme-ultraviolet radiation with a resolution of $\lambda/380$. *Nat. Phys.* **7,** 705–708 (2011).

[S3] Shwartz, S. & Harris, S. E. Polarization entangled photons at X-ray energies. *Phys. Rev. Lett.* **106,** 080501 (2011).

[S4] Shwartz, S. *et al.* X-ray parametric down-conversion in the Langevin regime. *Phys. Rev. Lett.* **109,** 013602 (2012).
